# Impact of Gastrointestinal Digestion Simulation on the Formation of Angiotensin-I-Converting Enzyme Inhibitory (ACE-I) Peptides from Germinated Lamtoro Gung Flour

**DOI:** 10.3390/foods11233769

**Published:** 2022-11-23

**Authors:** Aprilia Fitriani, Retno Indrati, Yustinus Marsono, Supriyadi Supriyadi

**Affiliations:** 1 Department of Food and Agricultural Products Technology, Faculty of Agricultural Technology, Universitas Gadjah Mada, Flora Street 1, Depok, Sleman 55281, Special District of Yogyakarta, Indonesia; 2 Food Technology, Faculty of Industrial Technology, Universitas Ahmad Dahlan, Jenderal Ahmad Yani Street, Banguntapan, Bantul 55166, Special District of Yogyakarta, Indonesia

**Keywords:** ACE-I peptides, lamtoro gung, gastrointestinal digestion simulation

## Abstract

The germination of lamtoro gung has been shown to increase the angiotensin-I-converting enzyme inhibitory (ACE-I) activity in previous studies. The 48 h germinated flour had the highest ACE-I activity. Administration of the gastrointestinal digestion (GID) simulation with commercial enzymes was expected to increase the ACE-I activity. However, the GID simulation to increase ACE-I in the germinated lamtoro gung flour has not been found. Therefore, this study aimed to evaluate the GID simulation of ACE-I peptides in sprouted lamtoro gung flour. This study also identified and characterised the peptide with the ACE-I activity. The GID simulation was performed using commercial pepsin (pH 2) and pancreatin (pH 7.5). Both simulations occurred at 37 °C for 240 min. The degree of hydrolysis, peptide concentration, and ACE-I activity was analysed. Samples with the highest ACE-I activity were then fractionated and identified, to determine the peptide responsible for the ACE-I activity. The 180 min GID simulation in the test sample showed the highest ACE-I activity (89.70%). This result was supported by an increased degree of hydrolysis (DH) and peptide concentrations throughout the GID simulation. The <1 kDa peptide fraction had the highest inhibitory activity and had the most elevated peptide portion (54.69%). Peptide sequences containing crucial amino acids were found in the <1 kDa peptide fraction. **P**RPPKP**P**, **P**PPPPGARA**P**, and **P**FPPSN**P**P**P** had proline in the C and N terminal residues. The peptides obtained also had other biological activities, such as a DPP IV inhibitor, an alpha-glucosidase inhibitor, and antioxidative activity. Based on the toxicity prediction, those peptides are non-toxic and safe to consume.

## 1. Introduction

Lamtoro gung (*Leucaena leucocephala* ssp. Glabrata (Rose) S. Zarate) belongs to the Fabaceae family and can be found in several tropical and subtropical countries [1]. Its protein content is approximately 24.5–46% [2]. The total negative-charge hydrophilic amino acids of lamtoro gung reach 13.97 g/100 g protein, and the total hydrophobic amino acids amount to 20.24 g/100 g protein [3]. These amino acid groups are essential for the utilisation of lamtoro gung peptides as an antihypertensive agent. The antihypertensive activity is evaluated by inhibiting the angiotensin-I-converting enzyme (ACE, E.C. 3.4.15.1) [4,5,6]. The inhibition can be demonstrated in vitro, in vivo, in situ, and in silico.

The negative-charge hydrophilic amino acids, such as glutamic acid and aspartic acid, can build an electrostatic interaction with the Zn^2+^ ions on the active site of ACE. In addition, the hydrophobic amino acids can form hydrophobic interactions with hydrophobic amino acid residues on the active site of ACE. These two interactions can potentially disturb the ACE structure and prevent its reaction with the substrate [4,5,6,7,8]. Therefore, the ACE inhibition is associated with the hypertension prevention through the ACE-I peptide consumption, given that ACE is responsible for blood vessel vasoconstriction, by converting angiotensin I (inactive form) to angiotensin II (active form) [8,9]. These conditions increase the risk of hypertension, coronary heart disease, and stroke.

The peptide size is vital in determining the ACE-I affinity, considering the extremely deep location of the ACE active site in the enzyme structure. The smaller the size of the peptide, the higher its capability to inhibit the ACE activity [4,5,10,11]. Increasing the peptide size will decrease the accessibility of the peptide to the active site of the enzyme [8]. Chickpea peptide with a molecular weight (MW) of 900 Da, showed a half maximal inhibitory concentration of 0.1 mg/mL toward the ACE-I activity [12]. The <3 kDa peptide fraction of pigeon peas showed a 86.65% ACE-I activity, significantly higher than that of the 3–10 kDa peptide fractions [13].

Peptides with a low MW can be obtained through hydrolytic processes, one of which is germination. Endopeptidase and exopeptidase hydrolyse the storage proteins, simultaneously [13]. The germination process increases the chickpeas’ low-MW peptides and ACE-I activity [14]. In a previous study (ongoing to the publish data), the germinated lamtoro gung flour produced from a 48 h germination showed similar results. In addition, the concentration of the low-MW peptides can be upgraded through the hydrolytic process using digestive enzymes to increase the ACE-I activity [10,15,16]. Commercial pepsin and pancreatin can be used to simulate the gastrointestinal digestion (GID) [17].

The amino acid profile of the germinated lamtoro gung flour, which is suitable to be used as a source of ACE-I peptides, is an opportunity to utilise them as a source of bioactive peptides. However, the utilisation of lamtoro gung as a source of ACE-I peptides has not been reported. Research on the germination of lamtoro gung for the production of ACE-I peptides has been carried out [18]. Unfortunately, the effect of the GID simulation on the ACE-I peptides formed has not been evaluated. Therefore, this study serves as follow-up research to gain comprehensive data on the profile of ACE-I peptides in germinated lamtoro gung flour during GID simulation. This study evaluated the GID simulation of ACE-I peptides in germinated lamtoro gung flour. The sample with the highest ACE-I activity was then fractionated through a dialysis membrane (1, 3.5, and 14 kDa MW cut-off (MWCO)). Each fraction was evaluated for its ACE-I activity. 

## 2. Materials and Methods

### 2.1. Materials

The germinated lamtoro gung flour came from a previous study (produced following the patent P00202103659). The other materials were distilled water, HCl, bovine serum albumin (SIGMA-A2153), pepsin (SIGMA-77161) and pancreatin (SIGMA-P1750), NaOH, phosphate buffer (pH 8), Folin–Ciocalteu’s phenol reagent (SIGMA-F9252), O-phthaldialdehyde (OPA) (SIGMA-P0657), β-mercaptoethanol, tryptophan (SIGMA-51145), sodium dodecyl sulfate (SDS) (SIGMA-L3771), ACE from a rabbit lung (SIGMA-A6778), hippury-histidyl -leucine (HHL) (SIGMA-H1635), captopril, Spectrum^TM^ Spectra/Por^TM^ 6 pre-wetted standard regenerated cellulose (RC) dialysis tubing (1 kDa (Cat No. 08-670-12C)), Spectrum^TM^ Spectra/Por^TM^ 3 RC dialysis tubing (3.5 kDa (Cat No. 08-670-5B)), and Spectrum^TM^ Spectra/Por^TM^ 4 RC dialysis tubing (14 kDa (Cat No. 08-66-7D)). In addition, some of the equipment used, included an analytical balance, vortex, electric stove, hot-plate stirrer, centrifuge, water bath shaker, spectrophotometer, and some glassware from Pyrex and Iwaki.

### 2.2. Peptide Extraction

The peptide extraction was performed following previous methods [19]. First, the germinated lamtoro gung flour was macerated in distilled water (1:10, *w*/*v*) at 30 °C for 1 h and centrifuged (3000× *g*; 4 °C) using Sorvall^TM^ ST 8 Centrifuge (Thermo Fisher Scientific, Waltham, MA, USA) for 20 min. Next, the supernatant, the peptide extract from the germinated lamtoro gung flour, was separated. Finally, the peptide extract was subjected to a GID simulation. 

### 2.3. GID Simulation

The digestion simulation was carried out following the work of Minekus et al. [17]. The peptides from the germinated lamtoro gung were gradually hydrolysed using pepsin and pancreatin. Briefly, aliquots of a 2 mL (5 mg/mL protein) sample were conditioned at pH 2 by adding 1 M HCl. Aquadest was used as a blank. The pepsin (EC. 3.4.23.1) (2000 U/mL) was added to the mixture. The mixture was incubated at 37 °C for 2 h in a water bath shaker (Memmert Water bath WNB 29, Germany). The addition of 0.9 M NaHCO_3_ (pH 5.3) terminated the reaction. The 2 M NaOH was used until pH 7.5 was reached. Pancreatin (100 U/mL) was added for the peptide hydrolysis simulation in the small intestine. The mixture was incubated at 37 °C for 2 h in a water bath shaker. Every 30 min during digestion, the sample was collected for further analysis. 

### 2.4. Degree of Hydrolysis (DH), Peptide Concentration Assay, and the MW Distribution Calculation

The DH and peptide concentration were evaluated in accordance with the work of Charoenphun et al. [20]. The OPA solution was prepared by dissolving 40 mg OPA to 1 mL methanol and 100 µL β-mercaptoethanol. Then, 25 mL 100 mM sodium tetra borate (Na_2_B_4_O_7_) and 2.5 mL 20% sodium dodecyl sulphate (SDS) were added to the OPA solution. Distilled water was added and adjusted to 50 mL with a volumetric flask.

A 3 mL OPA solution was added to a 0.4 mL prepared sample. The mixture was incubated at room temperature for 20 min (dark conditions). The absorbance was read at 340 nm using a spectrophotometer (Thermo Fisher Scientific GENESYS 10S UV-Visible Spectrophotometers, Waltham, MA, USA). Tryptophane (0.25 mg/mL) was used as a standard stock solution to obtain the amine concentration of each sample. The DH was calculated using Equation (1):

(1) $$\%\;\mathrm{DH}=\frac{\left(\left({\mathrm{NH}}_{2}\right)\mathrm{Tx}-\left({\mathrm{NH}}_{2}\right)T0\right)}{\left(\left({\mathrm{NH}}_{2}\right)\mathrm{Tot}-\left({\mathrm{NH}}_{2}\right)T0\right)}\times 100$$

where (NH_2_)Tx is the hydrolyzed sample at time x (test sample), (NH_2_)T0 is the hydrolyzed sample at 0 h, and (NH_2_)Tot is the fully hydrolyzed sample.

A fully hydrolysed sample was obtained by mixing a 250 mg sample with 10 mL 6 M HCl, containing 0.5% phenol for 4 h at 110 °C. Next, the sample was neutralised with 10 mL 6 M NaOH and centrifuged (3000 rpm, 15 min). Finally, the remaining supernatant was used.

The peptide concentration of the sample was obtained from the value of NH_2_ Tx and in consideration of the dilution factor, volume solvent, and sample weight. First, the MW distribution was calculated, based on the peptide concentration. Then, the MW distribution of each fraction was calculated using Equation (2):

(2) $$\%\;\mathrm{MW}\;\mathrm{distribution}\;x\;\mathrm{fraction}\;=\frac{\mathrm{Peptide}\;\mathrm{concentration}\;\mathrm{of}\;x\;\mathrm{fraction}}{\mathrm{Total}\;\mathrm{peptide}\;\mathrm{concentration}\;\mathrm{of}\;\mathrm{all}\;\mathrm{fraction}}\times 100$$

where the x fraction is the specific peptide fraction to be calculated.

### 2.5. ACE-I Activity Assay

The ACE-I activity assay was performed following the work of Cushman and Cheung [11]. The supernatant from the GID simulation was subjected to an ACE-I peptide. Briefly, aliquots of 50 µL samples (1 mg/mL) and 50 µL substrate solution (50 mM HHL) were mixed in a 2 mL Eppendorf tube. Distilled water was used as the blank and control. Captopril (1 mg/mL) was used as a positive control. Initially, 200 µL 1 M HCl was added to the blank. The mixture was incubated (37 °C; 10 min). Then, a 50 µL ACE solution (92.5 mU/mL) was added and incubated (37 °C; 30 min). Finally, 1 M HCl (200 µL) was added to stop the reaction. The product of the enzymatic reaction of ACE was hippuric acid. It was extracted by 1.5 mL ethyl acetate through vortex shaking at maximum speed. The mixture was centrifuged (14,000× *g*; 20 min). The supernatant (1 mL) was removed and placed in reaction tubes. Each reaction tube was placed in boiling water to evaporate the solvent until dryness (±15 min). Then, 3 mL bidistilled water was added and shaken. The absorbance of the solution was read at 228 nm with a spectrophotometer (Thermo Fisher Scientific GENESYS 10S UV-Visible Spectrophotometers, Waltham, MA, USA). The ACE-I activity of the sample was estimated using Equation (3):

(3) $$\%\;\mathrm{ACE}-I\;\mathrm{activity}=\frac{\left(A-B\right)}{\left(A-C\right)}$$

where A is the absorbance of the control solution, B is the absorbance of the sample solution, and C is the absorbance of the blank solution.

### 2.6. Peptide Fractionation Using the MW Cut Off (MWCO) Filtration

Peptide fractionation was used to obtain the peptide fraction with the highest ACE-I activity. Therefore, the peptide extract from the GID simulation was analysed for its ACE-I activity. The most increased ACE-I activity was fractionated through the RC filter membrane (1, 3.5, and 14 kDa MWCO). The pre-wetted 1 kDa RC membrane is a ready-to-use membrane. The dried RC membranes (3.5 and 14 kDa) were cleaned using boiled 10 mM NaHCO_3_ and 10 mM Na_2_EDTA for 10 min. The membrane was immersed in the boiled 10 mM Na_2_EDTA solution for 10 min. Finally, it was moved to the bidistilled water and considered ready for use. 

First, the peptide solutions were run through a 1 kDa membrane. Both ends of the membrane were closed tightly with a membrane clamp. Next, the membrane was placed in a beaker glass containing Aqua pro Injection. A magnetic stirring rod was inserted into a glass beaker. The beaker glass was placed on a plate stirrer and stored at a cold temperature (4 °C) during the fractionation process (12 h). The peptide solution was released on the Aqua pro Injection as <1 kDa peptide fraction. The remaining solution was then moved to the 3.5 kDa membrane, using the same procedures, and the last was used for the 14 kDa membrane. Thus, these step produced four peptide fractions: <1, 1–3.5, 3.5–14, >14 kDa. Each fraction was used to analyse the peptide concentration, peptide MW distribution, and the ACE-I activity.

### 2.7. Amino Acid Composition

The amino acid composition was determined using LC-MS (Thermo Fisher Scientific, Waltham, MA, USA). To begin, 2 mL of the fractionated peptide extract was put in a 50 mL screw test tube and adjusted the volume with Aqua pro Injection. Next, 20 mL mL HCl 6 N was added and hydrolysed (110 °C; 12 h). Then, 6 N NaOH was used to neutralise the solution and adjust the volume with Aqua pro Injection to 50 mL. It was diluted and filtered (0.22 µM). A 5 µL solution was injected into LC-MS. Elution was performed by using a mobile phase composed of solvent A (0.1% pentadecafluorooctanoic acid (PDFOA): water; 99.5%:0.5%) and solvent B (0.1% PDFOA: water; 10%:90%), at a flow rate of 0.6 mL/min. The gradient elution system was 0 min, 95% A; 5 min, 50% A; 5.20 min, 95% A; and 7 min, 95% A, and the temperature of the analysis was 25 °C. 

### 2.8. Characterisation of the Peptides and the Sequence Identification 

The fractionated peptide extract was analysed using LC-MS (Nano LC Ultimate 3000 series system tandem Q exactive™ plus orbitrap HRMS, Thermo Fisher Scientific, Waltham, MA, USA). The fractionated peptide extract was dissolved with Aqua pro Injection:acetonitrile (8:2, *v*/*v*). A 50 µL solution was injected into LC-MS. The mobile phase used was solvent A (water and 0.1% formic acid) and solvent B (acetonitrile and 0.1% formic acid), at a flow rate of 300 nL/min. The gradient elution system was 0–2 min, 2% B; 3–40 min, 2–99% B; 40–55 min, 99% B; 55–60 min. Proteome discoverer 2.2 software was used to identify the peptide sequence. The generated peptide sequence was then checked for the homology with https://www.uniprot.org/ and https://blast.ncbi.nlm.nih.gov/Blast.cgi, accessed on 4 November 2022.

### 2.9. Biological Potential of the Peptides

The biological potential of the fractionated peptide extract was studied, employing the BIOPEP-UWM database (https://biochemia.uwm.edu.pl/biopep-uwm/, accessed on 1 September 2022) [21]. The biological potential of the peptide sequences was evaluated based on the A (the frequency of the bioactive fragments’ occurrence in a peptide sequence) and B (potential biological activity of the protein fragments) values. Then, each peptide was checked for its binding model scheme with ACE, through the scheme proposed by Fan [7]. The peptides were analysed for their toxicity prediction, using ToxinPred database (https://webs.iiitd.edu.in/raghava/toxinpred/, accessed on 21 September 2022) [22]. 

### 2.10. Statistical Analysis

All data were expressed as mean values ± standard deviations (SDs) (*n* = 3). The IBM^®^ SPSS^®^ statistics (version 22, International business machines (IBM) corporation, Armonk, NY, USA) was used. A statistical comparison used a one-way analysis of variance (ANOVA) and an independent sample *t*-test with differences between means at the 5% (*p <* 0.05) level, considered significant.

## 3. Results

### 3.1. %DH and the Peptide Concentration of the Germinated Lamtoro Gung Flour during the GID Simulation

**Figure 1:**
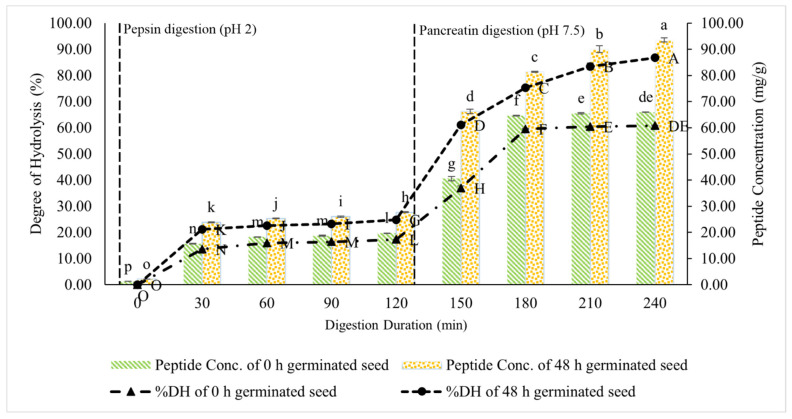
%DH and the peptide concentration of the germinated lamtoro gung flour at different digestion durations. Values are means with their SDs. Capital letters indicate significant differences between the samples of the %DH, and the small letters indicate significant differences between the samples of the peptide concentration (Duncan’s test, α = 0.05).

The percentage of the DH and the peptide concentration were analysed to investigate the increased ACE-I activity. The %DH explains how the hydrolysis created the small peptides that contributed to the ACE-I peptides. Figure 1 shows the %DH and peptide concentration of the germinated lamtoro gung flour during the GID simulation.

The %DH is presented as a line curve, and the peptide concentration is a column curve. The 0 (control sample) and the 48 h (test sample) germinated seed significantly increased the pancreatic digestion phase. The percentage of the DH from the control sample was 36.96% at 150 min digestion and reached up to 60.81%, at the end of the digestion period. These values aligned with the peptide concentration, whose values ranged from 40.58 mg/g (150 min) to 65.96 mg/g (240 min). The test sample showed a higher %DH and peptide concentration. The %DH of the test sample increased from 61.03% (150 min) to 86.76% (240 min). The peptide concentration increased from 66.2 mg/g (150 min) to 93.57 mg/g (240 min).

### 3.2. ACE-I Activity of the Germinated Lamtoro Gung Flour during the GID Simulation

**Figure 2:**
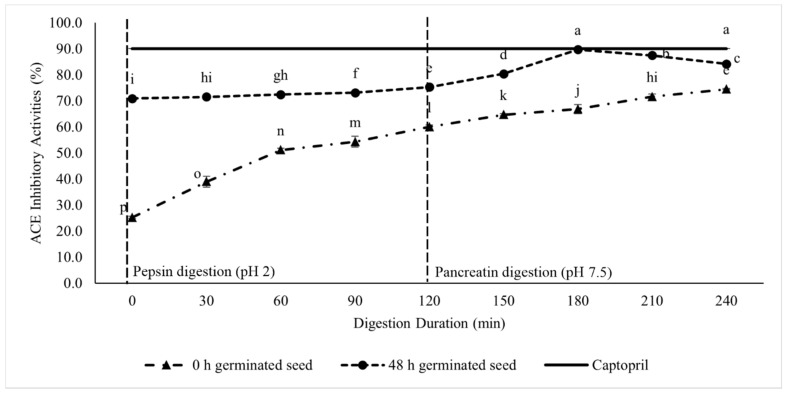
ACE-I activity of the germinated lamtoro gung flour at different digestion durations. Values are means with their SDs. Different letters indicate significant differences between the samples (Duncan’s test, α = 0.05).

The ACE-I activity was evaluated in each part of the digestion simulation. The first was for the pepsin digestion, and the last was for the sequential digestion of pepsin and pancreatin. Figure 2 displays the ACE-I activity of the germinated lamtoro gung flour during the GID simulation. During the pepsin digestion, the control group gradually exhibited an increased ACE-I activity. For example, it was 25.24% at 0 min digestion and reached up to 59.96% at 120 min. Moreover, the 48 h germinated seed showed a low increment of about 70.92% inhibition to 75.28% at 120 min digestion.

The control sample gradually improved during the pancreatin digestion until digestion ended (240 min). However, the test sample (48 h germinated flour) showed a significant increase at 150 min (80.38%) to 180 min (89.70%) of digestion and then decreased. The digestion simulation for 180 min resulted in the highest ACE-I values, among the samples, and such a result was not significantly different from that of the positive control sample (captopril). Thus, this sample was selected for the peptide fractionation process.

### 3.3. Peptide Fractionation

Based on the highest ACE-I activity observed during the 180 min GID simulation, compared with the other samples, the 48 h germinated seed was selected for the fractionation process through the membrane dialysis procedures. The control sample was also fractionated. Three MWCO membranes were used and resulted in four peptide fractions: the <1, 1–3.5, 3.5–14, and >14 kDa peptide fractions. Each fraction was evaluated for its peptide concentration, MW distribution, and ACE-I activity. 

#### 3.3.1. Peptide Concentration of Each Fraction

**Figure 3:**
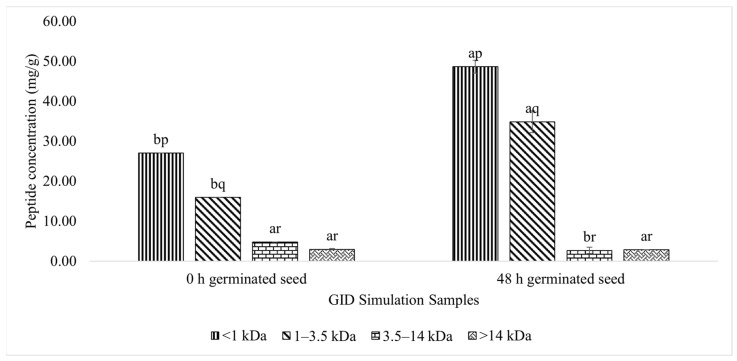
Peptide concentration of the germinated lamtoro gung flour at different fractions after the GID simulation. Values are means with their SDs. a and b indicate significant differences between the same fraction peptide in different samples (*t*-test, α = 0.05). p, q, and r indicate significant differences between the different peptide fractions in the same sample (Duncan’s test, α = 0.05).

The concentration of every peptide fraction was analysed, to obtain the peptide concentration (Figure 3). The <1 kDa peptide fraction had the highest concentration in the test and control samples, followed by the 1–3.5 kDa fraction. The 3.5–14 and >14 kDa peptide fractions showed lower concentrations, which were affected by hydrolysis during the GID simulation.

The control sample contained 27.04 and 15.92 mg/g peptide in the <1 kDa and 1–3.5 kDa fractions, respectively. In addition, the test sample had 48.65 and 34.83 mg/g peptide in the <1 and 1–3.5 kDa fractions, respectively. These amounts were considerably higher than those of the control sample.

#### 3.3.2. MW Distribution of Each Fraction

**Figure 4:**
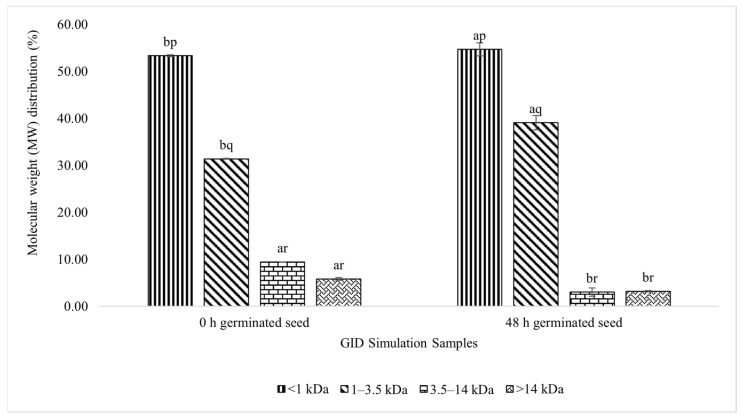
MW distribution of the germinated lamtoro gung flour peptide in different fractions after the GID simulation. Values are means with their SDs. a and b letters indicate significant differences between the same peptide fraction in different samples (*t*-test, α = 0.05). p, q, and r indicate significant differences between the different peptide fractions in the same sample (Duncan’s test, α = 0.05).

The MW distribution of the peptide fractions (Figure 4) showed the percentage of certain peptide fractions in the complete peptide solution. In line with the peptide concentration results, <1 kDa fraction of both samples had a higher distribution than the others. The distribution of the <1 kDa peptide fraction for the control and test samples were 53.35% and 54.69%, respectively. These distributions were more than 50%, compared with the other fractions. These results explain that <1 kDa fraction had the highest portion in each sample. 

#### 3.3.3. ACE-I Activity of Each Peptide Fraction

**Figure 5:**
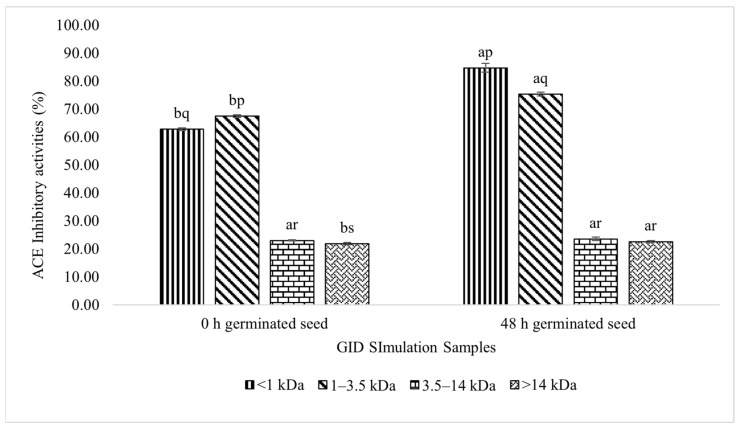
ACE-I activity of the germinated lamtoro gung flour peptide in different fractions after the GID simulation. Values are means with their SDs. a and b indicate significant differences between the same peptide fraction in different samples (*t*-test, α = 0.05). p, q, r, and s indicate significant differences between the different peptide fractions in the same sample (Duncan’s test, α = 0.05).

The ACE-I activity analysis was performed on each peptide fraction to obtain the peptide fraction with the highest ACE-I activity (Figure 5). The <1 kDa peptide fraction of the test samples had the highest increase in the inhibition activity (84.72%). In addition, the 1–3.5 kDa peptide fraction had the highest ACE-I activity in the control samples. Among the control and test samples, the >14 kDa peptide fraction had the lowest inhibition activity.

#### 3.3.4. Amino Acid Composition

The <1 kDa and 1–3.5 kDa peptide fractions, which had a high ACE-I activity, were then analysed for the amino acid composition (Table 1). This analysis aims to determine the concentration of the amino acid composition retained during the fractionation process with a dialysis membrane. Both fractions showed similarities in the amino acid types with varying concentrations. Based on the analysis results obtained, there are 10 types of amino acids detected and following the limit of detection (LOD) of the instrument used.

#### 3.3.5. Identification of the Lamtoro Gung Flour Peptide and its Inhibitory Effect

The <1 and 1–3.5 kDa peptide fractions obtained from the GID simulation were then sequenced. The <1 kDa fraction resulted in 18 sequenced peptides, and 11 peptides had high B values (potential biological activity of the protein fragments), which are presented in Table 2. The MW of the eight peptides ranged from 787.47–998.66 Da. The B values were generated from the peptide sequence simulation using the BIOPEP-UWM database and accompanied by the toxicity predictions using ToxinPred. Based on the results of the BIOPEP-UWM simulation, the resulting peptide showed an inhibitory activity against ACE. In addition, the peptides exhibited other biological activities, such as the inhibition of dipeptidyl peptidase IV (DPP-IV) and alpha-glucosidase, related to the glucose metabolism and also the antioxidant activity. Based on the simulation results of the peptide toxicity, all peptide sequences showed predictions of non-toxin peptides. This means that the peptides generated from the GID simulation of the germinated lamtoro flour are safe. 

The fractions of 1–3.5 kDa produced 158 sequenced peptides, and six peptides had high B values, which are presented in Table 3. The MW of them ranged from 1037.62–1786.90 Da. Based on the results of the BIOPEP-UWM simulation, the resulting peptide showed an inhibitory activity against ACE. In addition, the peptide also showed other biological activities, similar to the <1 kDa fraction and the predictive non-toxin peptides.

## 4. Discussion

The DH indicates the percentage of the breakdown of the high molecular weight (MW) peptides into low MW peptides. The increase in the %DH occurred in the first 30 min of the pepsin hydrolysis phase, in the control and test samples. However, until the 120th minute, each sample showed no significant increase. An increase in the %DH was found in the GID simulation of the pancreatin phase, which was 150 min, until the final stage. The %DH significantly increased in both samples, although the test sample showed a higher value. A similar trend was also observed in the peptide concentration data. The peptide concentration was in line with the %DH.

The difference in the %DH and the peptide concentration in the pepsin and pancreatin phases was due to the different enzymes used. Pancreatin consists of several protease enzymes, such as trypsin, α-chymotrypsin, and carboxypeptidase [23]. Thus, pancreatin exhibits several protein-cleavage capabilities, including trypsin-like activity, which breakdown protein in cationic amino acids, such as Arg and Lys, chymotrypsin-like activity, which cleaves aromatic amino acids and branched-chain amino acids (Tyr, Phr, Trp, Leu), carboxypeptidase activity which cleaves all amino acid residue sites except Asp, Glu, Arg, and Lys, and the elastase activity which cleaves all Ala sites [10,16,24]. The pancreatin digestion can produce a broader breakdown, compared with the use of pepsin. These results are consistent with the GID simulation studies on the pigeon pea tempe [25], pigeon pea protein isolate [16], and the jack bean tempe [10].

The increases in %DH and the peptide concentrations are closely related to the activity of the ACE-I produced. Based on previous research, the small MW is one of the characteristics of ACE-I peptides [7,8]. In addition, the hydrolysis that occured during the GID simulation supported the formation of the low MW peptides, which can contribute to the increase in the ACE-I activity [10,15,16]. This finding was indicated by the rise in the ACE-I activity during the GID simulation in the pepsin and pancreatin digestion phases. The test sample showed a significantly higher ACE-I activity, compared with the control sample. The GID simulation at 180 min showed the most increased ACE-I activity in the test sample. However, this result was not significantly different from that of the positive control sample (captopril). 

In addition, the activity of ACE-I during the hydrolysis process, was influenced by the protein substrate, proteolytic enzymes used, and the hydrolysis conditions. These factors affect the structure and sequence of the bioactive peptides contained in protein hydrolysates [26]. The test sample at 180 min of the GID simulation had the highest ACE-I activity, due to the formation of peptides with the size, the amino acid composition, and the amino acid sequence matching those of the ACE inhibitory peptides. Similar results were also found in previous studies [10,16,19,25].

The 180 min GID simulation sample with the highest ACE-I inhibitory activity was then fractionated through a dialysis membrane with three types of MWCO. Based on this process, four peptide fractions were produced, namely, <1, 1–3.5, 3.5–14, and >14 kDa fractions. Figure 3 shows the peptide concentration obtained for each fraction. The GID simulation showed the breakdown of the high MW peptides (3.5–14 and >14 kDa) into peptides with a low MW (1–3.5 and <1 kDa). The <1 kDa fraction had the highest peptide concentration. These results support the data on the samples’ %DH and the peptide concentrations during the GID simulation. The increase in the concentration of the low MW peptides and the decrease in the concentration of the high MW peptides resulted from the hydrolysis process that occured during the GID simulation [5].

Figure 4 shows the proportions of each peptide fraction. Based on the resulting peptide concentration data, the MW distribution data were obtained, and they showed the distribution of each peptide fraction in each sample. In the test and control samples, the <1 kDa peptide fraction had the highest portion, compared with the other fractions. This finding follows the previously described results of the peptide concentrations in each fraction.

The ACE-I activity in each fraction was evaluated to determine the peptide fraction that contributed to the inhibition of the ACE-I peptides from the germinated lamtoro gung flour. In the control and test samples, the <1 and 1–3.5 kDa fractions attained high ACE-I inhibitory values. The high ACE-I activity of the <1 kDa fraction followed the previously described results [8,10,12,26], which indicated that the peptide fractions with a small MW can exhibit an ACE-I activity. This property is related to the location of the ACE active site in deep and narrow areas, in which small peptides are required to access the ACE active site and prevent its catalytic reaction with the substrate [8,27].

Nevertheless, the <1 kDa fraction in the test sample showed the highest and most significant ACE-I activity, compared with other peptide fractions. The <1 kDa fraction of the test sample was significantly higher than that of the control sample. These results showed that the ACE-I activity is also affected by the amino acid sequence. The ACE-I peptide from the <1 kDa fraction was expected to consist of amino acid residues that are essential in inhibiting the ACE activity [7]. This result follows a finding on the pigeon pea protein hydrolysate [16], that the <3 kDa peptide fraction had the highest ACE-I activity than the higher peptide fraction.

Referring to the peptide binding scheme with ACE, proposed by Fan [7], the C terminal amino acid residue at the P2’ position binds to the ACE amino acid residue on the S2’ subsite. The peptide at the P1’ position binds to the S1’ subsite and the peptide at the P1 position binds to the S1 subsite. Table 2 and Table 3 show the peptide sequence that plays a role in inhibiting the ACE activity.

The presence of a proline (P) at the P2’ position has two roles. First, the carboxyl group contributes to forming hydrogen bonds with the −OH group of Tyr520 and the amine group of Gln281 [28]. Second, the pyrrolidine ring on the proline side chain can form strong hydrophobic interactions with aromatic amino acid residues on the S2’ subsite [7,9,28]. In addition, lysine (K) and arginine (R), as positively charged polar amino acids, also play an essential role at the C terminal, because they can build electrostatic interactions with the carboxyl group of the amino acid residues on the active site of ACE [29].

The positively charged polar amino acids (K and R) at the P1’ position (penultimate position of the C terminal) can form electrostatic interactions with the carboxyl group of Glu162 at the S1’ [7]. Aliphatic amino acids, such as valine (V) and leucine (L) are necessary for the P1’ position to form hydrophobic interactions with the hydrophobic amino acid residues at S1’ [30]. 

The S1 subsite on the active site of ACE consists of hydrophobic amino acid residues, such as Ala354, Val518, Phe512, and others. Therefore, the presence of the hydrophobic amino acid residues, such as isoleucine (I), valine (V), and proline (P) at the P1 position (3rd order of the C terminal peptide) is essential. Those amino acid residues built the hydrophobic interaction and the hydrogen bonds at the active site of ACE, thereby triggering a conformational change in the amino acid residue bound to Zn^2+^ [7]. Leucine (L) has a more hydrophobic potential than isoleucine because leucine has a methyl group branch at Cγ, compared to isoleucine which only has one methyl group at Cγ. The presence of two methyl groups increases the binding ability of the S1 subsite [7,9]. Aliphatic and hydrophobic amino acids are essential for the N terminal [30]. The valine (V) at the N terminus binds to the His353 and His513 side chains at the ACE active site via the hydrogen bonding and hydrophobic interactions [28]. Based on Table 1, <1 kDa peptide sequenced has lysine, arginine, and isoleucine in high concentrations. This follows the resulting peptide sequence data (Table 2). At the same time, the 1–3.5 kDa peptide fraction found high amounts of lysine and leucine. This is also in line with the discovery of the peptide sequences (Table 3).

## 5. Conclusions

The GID simulation can increase the %DH and peptide concentration of the germinated lamtoro gung flour. The digestion of the pancreatin phase showed a higher %DH than pepsin. The GID simulation for 180 min showed the highest ACE-I activity, compared with other GID simulation durations. The increase in %DH during the GID simulation resulted in high peptide concentrations in the low MW peptide fraction, with a peptide distribution exceeding 50% of the total peptide portion. In addition, the <1 and 1–3.5 kDa peptide fractions showed a high ACE-I activity, proving that the low MW peptides can exhibit a biological activity that inhibits the ACE activity.

Several peptide sequences containing crucial amino acids were found in the <1 kDa peptide fraction. **P**RPPKP**P**, **P**PPPPGARA**P**, and **P**FPPSN**P**P**P** had proline in the C and N terminal residue. The IAGLDV**K**R and KAGQL**R**K peptides contain lysine and arginine, respectively at the P1’ position. The hydrophobic amino acid residue at the P1 position was found on SLEGG**I**PR, SKIVKV**I**GR, KAGQ**L**RK, LVNPT**I**PR, PFPPSN**P**PP, IAGLD**V**KR, TAPPPPP**P**PK, and PLELV**G**LR peptides. Other biological activities, such as the DPP IV inhibitor, an alpha-glucosidase inhibitor, and antioxidative activity were also found on the peptides. Those activities need to be explored for future research. Based on the toxicity prediction, those peptides are non-toxic and safe to consume. The limitation of this research is that the peptide database for the lamtoro gung species has not been found, so the peptides produced in the identification process are only found to be homologous to peptides from other legumes.

## Figures and Tables

**Table 1:** Amino acid composition of the peptide fraction.

No	Amino Acids	Concentration (mg/L)	
		<1 kDa Peptide Fraction	1–3.5 kDa Peptide Fraction
1	L-arginine (R)	102 ^a^ ± 4	28.1 ^b^ ± 0.3
2	L-histidine (H)	322 ^a^ ± 1	102 ^b^ ± 1
3	L-lycine (K)	881 ^a^ ± 15	331 ^b^ ± 1
4	L-phenylalanine (F)	86 ^a^ ± 4	90 ^a^ ± 1
5	L-isoleucine (I)	100 ^a^ ± 1	(699.05 ^b^ ± 0.04) × 10^−1^
6	L-leucine (L)	85 ^b^ ± 2	120.4 ^a^ ± 0.3
7	L-proline (P)	(0.1 ± 0.1) × 10^−2^	n.d.
8	L-glycine (G)	(0.2 ± 0.1) × 10^−2^	n.d.
9	L-thryptophan (W)	0.1 ^a^ ± 0.1	0.02 ^a^ ± 0.02
10	L-tyrosine (Y)	n.d.	(0.03 ± 0.02) × 10^−2^

Note: Numbers with their SDs followed by the different letters within the same row indicate a significant difference (*t*-test, α = 0.05). n.d. = not detected.

**Table 2:** Peptide sequence of the germinated lamtoro gung flour obtained from the GID simulation <1 kDa peptide fraction.

No	Peptide Sequence	MW (Da)	Toxicity Prediction	Activity	The Frequency of the Bioactive Fragments (A)	Potential Biological Activity of the Protein Fragments (B)	Master Protein	Accession Number
1	**P**RPPKP**P**	787.47	Non-toxin	ACE inhibitor	1	0.04220	OS = Glycine max	K7LI30
				Alpha glucosidase inhibitor	0.28	1.58000		
				DPP IV inhibitor	0.71	0.00016		
2	**P**PPPPGARA**P**	955.53	Non-toxin	ACE inhibitor	1.2	0.0056	Formin-like protein OS = Medicago truncatula	A0A396IL77
				Alpha glucosidase inhibitor	0.4	0.000022		
				DPP IV inhibitor	1.1	0.000125		
3	SLEGG**I**P**R**	827.46	Non-toxin	ACE inhibitor	0.625	0.03200	LRR receptor-like kinase resistance protein OS = Trifolium pratense	A0A2K3P9T5
				DPP IV inhibitor	0.625	0.00035		
4	SKIVKV**I**G**R**	998.66	Non-toxin	ACE inhibitor	0.3333	0.0087	CTP synthase OS = Lupinus albus	A0A6A5KTD9
5	**K**AGQ**LRK**	799.51	Non-toxin	ACE inhibitor	0.5714	0.00551	Pentatricopeptide repeat-containing protein, mitochondrial OS = Glycine soja	A0A0B2RHL1
				DPP IV inhibitor	0.5714	0.00002278		
6	**L**VNPT**I**P**R**	908.55	Non-toxin	ACE inhibitor	0.5	0.051	CCHC-type domain-containing protein OS = Arachis hypogaea	A0A445AQL8
				DPP IV inhibitor	0.75	0.000304		
7	**P**FPPSN**P**P**P**	948.47	Non-toxin	ACE inhibitor	0.8889	0.0035	FAS1 domain-containing protein OS = Vigna angularis var. angularis	A0A0S3QYH1
				Alpha glucosidase inhibitor	0.3333	0.0000184		
				DPP IV inhibitor	0.7778	0.000362		
8	**I**AGLD**VKR**	870.53	Non-toxin	ACE inhibitor	0.625	0.01080	OS = Lupinus albus	A0A6A5LEL5
				DPP IV inhibitor	0.625	0.00005		
9	THGHIQ**VK**	918.51	Non-toxin	ACE inhibitor	0.375	0.00967	OS = Arachis hypogaea	A0A444ZCU3
				DPP IV inhibitor	0.75	0.00255		
10	TAPPPPP**P**P**K**	997.57	Non-toxin	ACE inhibitor	1.4	0.03550	OS = Trifolium subterraneum	A0A2Z6MUR4
				Alpha glucosidase inhibitor	0.6	0.00003		
				DPP IV inhibitor	1.3	0.00012		
11	**P**LELV**GLR**	895.56	Non-toxin	ACE Inhibitor	0.5	0.001325	OS = Glycine soja	A0A445FEF3
				DPP IV inhibitor	0.625	0.0000478		

**Table 3:** Peptide sequence of the germinated lamtoro gung flour obtained from the GID simulation <1–3.5 kDa peptide fraction.

No	Peptide Sequence	MW (Da)	Toxicity Prediction	Potential Biological Activity	The Frequency of the Bioactive Fragments (A)	Potential Biological Activity of the Protein Fragments (B)	Master Protein	Accession Number
1	**V**APmSTGQATSERG**A**	1477.69	Non-toxin	ACE inhibitor	0.57	0.054120	Cellulose synthase OS = Phaseolus vulgaris	V7BSE0
				Dipeptidyl peptidase IV inhibitor	0.64	0.000434		
2	**K**DGLSPDHRTLSA**YI**D	1786.90	Non-toxin	ACE inhibitor	0.38	0.074970	OS = Arachis hypogaea	A0A444WW07
				Dipeptidyl peptidase IV inhibitor	0.38	0.000034		
3	**R**GLPVR**G**Q**R**	1037.62	Non-toxin	ACE inhibitor	0.78	0.072000	Adenylate kinase OS = Lupinus albus	A0A6A5LHC6
				Dipeptidyl peptidase IV inhibitor	0.67	0.000224		
4	**K**ATNSTAPEVNPR**LLK**	1737.97	Non-toxin	ACE inhibitor	0.56	0.061000	UV-stimulated scaffold protein A homolog isoform X1 OS = Abrus precatorius	A0A8B8L9K4
				alpha-glucosidase inhibitor	0.06	0.000002		
				Dipeptidyl peptidase IV inhibitor	0.63	0.000018		
5	**A**FLPGSLVDVRP**V**	1368.78	Non-toxin	ACE inhibitor	0.69	0.052600	30S ribosomal protein S1 OS = Lupinus albus	A0A6A5KWC6
				Dipeptidyl peptidase IV inhibitor	0.77	0.000383		
6	**G**PVLEDWEKDLGPPSG**G**	1751.85	Non-toxin	ACE inhibitor	0.65	0.097600	OS = Trifolium medium	A0A392UIN2
				alpha-glucosidase inhibitor	0.06	0.000003		
				antioxidative	0.12	0.000009		
				Dipeptidyl peptidase IV inhibitor	0.59	0.000909		

## Data Availability

Data are contained within the article.
